# Risk factors influencing the outcome of peptic ulcer bleeding in chronic kidney disease after initial endoscopic hemostasis: A nationwide cohort study

**DOI:** 10.1097/MD.0000000000004795

**Published:** 2016-09-09

**Authors:** Chih-Ming Liang, Chien-Ning Hsu, Wei-Chen Tai, Shih-Cheng Yang, Cheng-Kun Wu, Chih-Wei Shih, Ming-Kun Ku, Lan-Ting Yuan, Jiunn-Wei Wang, Kuo-Lun Tseng, Wei-Chih Sun, Tsung-Hsing Hung, Seng-Howe Nguang, Pin-I Hsu, Deng-Chyang Wu, Seng-Kee Chuah

**Affiliations:** a Division of Hepatogastroenterology, Department of Internal Medicine; b Department of Pharmacy, Kaohsiung Gang Gung Memorial Hospital, Kaohsiung; c School of Pharmacy, Kaohsiung Medical University, Kaohsiung; d Chang Gung University, College of Medicine, Kaohsiung; e Division of Hepatogastroenterology, Department of Internal Medicine, Chiayi Chang Gung Memorial Hospital, Chiayi; f Division of Gastroenterology, Fu-Ying University Hospital, Pin-Tung; g Divisions of Gastroenterology, Yuan General Hospital, Kaohsiung; h Division of Gastroenterology, Department of Internal Medicine, Kaohsiung Medical University Hospital, and Kaohsiung Municipal Ta-Tung Hospital, Kaohsiung; i Division of Gastroenterology, Department of Internal Medicine, Kaohsiung Veterans General Hospital, National Yang-Ming University, Kaohsiung; j Division of Gastroenterology; Department of Medicine, Dalin Tzu Chi Hospital, Buddhist Tzu Chi Medical Foundation, Chiayi; k Division of Gastroenterology; Pin-Tung Christian Hospital, Pin-Tung, Taiwan.

**Keywords:** chronic kidney disease, endoscopic treatment, nationwide cohort study, peptic ulcer bleeding, rebleeding

## Abstract

Patients with chronic kidney disease (CKD) who had peptic ulcer bleeding (PUB) may have more adverse outcomes. This population-based cohort study aimed to identify risk factors that may influence the outcomes of patients with CKD and PUB after initial endoscopic hemostasis. Data from 1997 to 2008 were extracted from the National Health Insurance Research Database in Taiwan. We included a cohort dataset of 1 million randomly selected individuals and a dataset of patients with CKD who were alive in 2008. A total of 18,646 patients with PUB were screened, and 1229 patients admitted for PUB after endoscopic hemostasis were recruited. The subjects were divided into non-CKD (n = 1045) and CKD groups (n = 184). We analyzed the risks of peptic ulcer rebleeding, sepsis events, and mortality among in-hospital patients, and after discharge. Results showed that the rebleeding rates associated with repeat endoscopic therapy (11.96% vs 6.32%, *P* = 0.0062), death rates (8.7%, vs 2.3%, *P* < 0.0001), hospitalization cost (US$ 5595±7200 vs US$2408 ± 4703, *P* < 0.0001), and length of hospital stay (19.6 ± 18.3 vs 11.2 ± 13.1, *P* < 0.0001) in the CKD group were higher than those in the non-CKD group. The death rate in the CKD group was also higher than that in the non-CKD group after discharge. The independent risk factor for rebleeding during hospitalization was age (odds ratio [OR], 1.02; *P* = 0.0063), whereas risk factors for death were CKD (OR, 2.37; *P* = 0.0222), shock (OR, 2.99; *P* = 0.0098), and endotracheal intubation (OR, 5.31; *P* < 0.0001). The hazard ratio of rebleeding risk for aspirin users after discharge over a 10-year follow-up period was 0.68 (95% confidence interval [CI]: 0.45–0.95, *P* = 0.0223). On the other hand, old age (*P* < 0.0001), CKD (*P* = 0.0090), diabetes (*P* = 0.0470), and congestive heart failure (*P* = 0.0013) were the independent risk factors for death after discharge. In-hospital patients with CKD and PUB after endoscopic therapy had higher recurrent bleeding, infection, and mortality rates, and the need for second endoscopic therapy. Age was the independent risk factor for recurrent bleeding during hospitalization. After being discharged with a 10-year follow-up period, nonaspirin user was a significant factor for recurrent bleeding.

## 1 Introduction

Taiwan is among the top 3 countries with the highest incidence of chronic kidney disease (CKD) in the world according to the 2010 report of the United States Renal Data System.^[1]^ Patients with CKD are at increased risk for peptic ulcer disease (PUD) than the general population in long-term follow-up.^[2,3]^ Furthermore, these data are relevant with regard to higher peptic ulcer bleeding (PUB) complications and mortality rate compared with the general population.^[4–6]^ Moreover, one of the predictors of mortality in validated upper gastrointestinal bleeding scoring systems, such as the Rockall score is renal insufficiency.^[7,8]^ Improvement of outcomes in PUB was attained only after the introduction of interventional endoscopic therapy and high-dose acid suppression.^[9–11]^ Reports on outcome studies of PUB in CKD patients after invasive endoscopic hemostasis are rare. In our previous study,^[12]^ patients with end-stage renal disease (ESRD) and CKD were associated with higher rebleeding rate and in-hospital mortality after endoscopic hemostasis in high stigmata ulcer bleeding than the control group. However, the data scale is small and limited to one tertiary hospital. In an attempt to overcome these limitations, we used the Taiwan National Health Research Institute database (NHRID; a cohort dataset for 1 million randomly selected individuals) to conduct a population-based case–control study aiming to determine the risk factors influencing the outcomes of patients with CKD and PUB after initial endoscopic hemostasis. We also determined the incidence of PUD over a 10-year follow-up period after initial endoscopic hemostasis between CKD and non-CKD groups.

## 2 Methods

**Figure 1:**
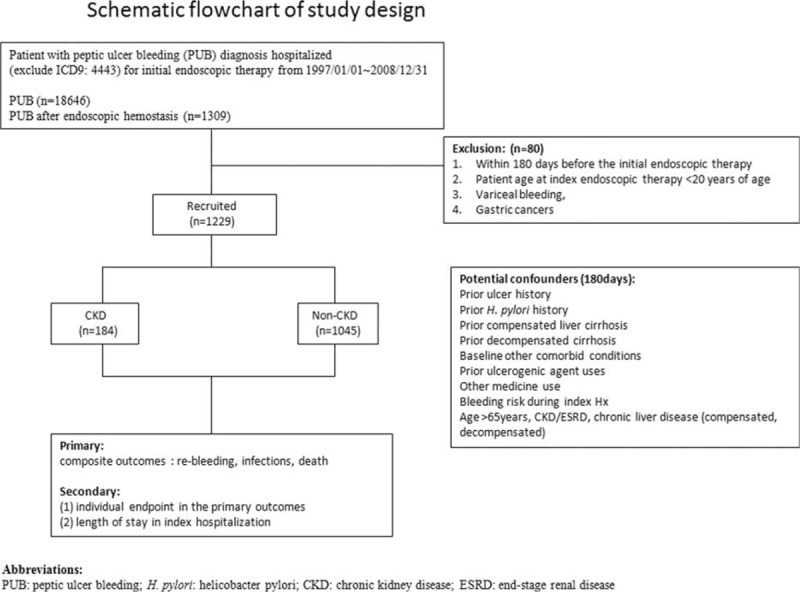
Schematic flowchart of the study design.

This protocol was approved by the institutional review board and the Ethics Committee of Chang Gung Memorial Hospital (IRB104-1343B). Claims data in the present study (from 1997 to 2008) were collected in Taiwan's NHIRD at the National Health Research Institutes who released a cohort dataset for 1 million randomly selected individuals and a dataset for patients with some severe illnesses who were alive until 2008. Kaohsiung Medical Center is one of the sites of the Collaboration Center of Health Information Application, Ministry of Health and Welfare. The data analyst in the present study was one of the staffs who analyzed the institute comprehensive health care data, which included the enrollment files, claims data, catastrophic illness files, and registry for drug prescriptions. The 9th Revision of the International Classifications of Diseases Codes (ICD-9 codes)were used to define diseases. Each patient's original identification number was deidentified. We screened all patients with first admission to the hospital with a primary diagnosis of PUB (ICD-9 codes: 531.0, 531.2, 531.4, 531.6, 532.0, 532.2, 532.4, 532.6, 533.0, 533.2, 533.4, and 533.6). Patients with PUB (n = 18,646) were enrolled as shown in Fig. 1. Among patients with PUB, those who were diagnosed with uremia were patients with CKD diagnosed on the previous admission (ICD-9 codes 585 and 586) or patients who underwent regular hemodialysis (more than 9 months) before the index PUB hospitalization, but not patients who underwent postrenal transplantation were initially enrolled (n = 1309). A total of 1229 patients were eventually recruited for analysis after we excluded 80 patients who were <20 years old, encountered PUB with endoscopic treatment within 180 days before index, had bleeding varices, had gastric resection or vagotomy, and those with gastric cancer who developed the disease within the first 2 years of the index hospitalization. We then divided the patients into 2 groups: non-CKD (n = 1045) and CKD groups (n = 184).

Among them, patients who underwent repeated endoscopic treatment (47043B) and needed surgery because of bleeding (72006B, 72007B, 72009B, 72010B, 72011B, 72012B, 72018B, and 72019B) were analyzed. Detailed data such as dose, frequency, starting and ending dates, and administration routes of the prescriptions of ulcerogenic drugs during the follow-up period after the index hospitalization were also obtained from the dataset. Aspirin, nonsteroid anti-inflammatory drugs (NSAIDs), cyclooxygenase 2-specific inhibitors, and other anticoagulants (clopidogrel, dipyridamole, warfarin, ticlopidine, cilostazol, and cerenin) were among these ulcerogenic drugs. Any comorbid conditions, such as previous ischemic heart disease, cerebral infarction, hypertension, diabetes, chronic obstructive lung disease, liver cirrhosis, and hyperlipidemia, diagnosed on admissions before the index hospitalization and during the follow-up period were included for analysis. Patients were considered as having *Helicobacter pylori*-associated peptic ulcer if a record of *H pylori* eradication therapy was identified during or after the index hospitalization such as proton pump inhibitors (PPIs) or H2 receptor antagonists (H2RA), plus clarithromycin or metronidazole, plus amoxicillin or tetracycline, with or without bismuth, and other regimens.^[13]^ We analyzed the risks of peptic ulcer rebleeding, sepsis events, mortality during hospitalization and after being discharged in this special population.

### 2.1 Statistical analysis

Descriptive statistics was applied to all variables. Continuous data were presented as means (standard deviation, SD) and median (interquartile range), and categorical data as actual frequencies and percentages. Baseline characteristics were compared using unpaired Student *t* test and chi-square analysis of contingency tables for continuous and nominal variables, respectively. Multivariate logistic regression was applied to examine factors associated with treatment allocation. Kaplan–Meier plot and Cox proportional hazards ratio were applied to compare the outcomes of interest between groups. Adjustments were made in the multivariate analysis for patient demographics, clinical conditions, and drug use. All *P* values were 2-tailed, and values <0.05 were considered statistically significant. All analyses were performed using the statistical software package SAS version 9.3 (SAS Institute Inc., Cary, NC, 2013).

## 3 Results

Table 1 shows the clinical characteristics of all patients. Significant differences were found between the CKD and non-CKD groups for age (68.15 ± 12.40 vs 62.39 ± 16.18, *P* < 0.0001), Charlson scores (2.76 ± 2.53 vs 1.22 ± 1.55, *P* < 0.0001), antibiotic use (47.28% vs 33.11%, *P* = 0.0002), hospital infections (26.63% vs 17.42%, *P* = 0.0032), and prior peptic ulcer history (10.87%, vs 4.02%, *P* < 0.0001). Ulcerogenic agent prescriptions, such as PPI or H2RA, 90 days before hospitalization were not significantly different between the 2 groups.

**Table 1:**
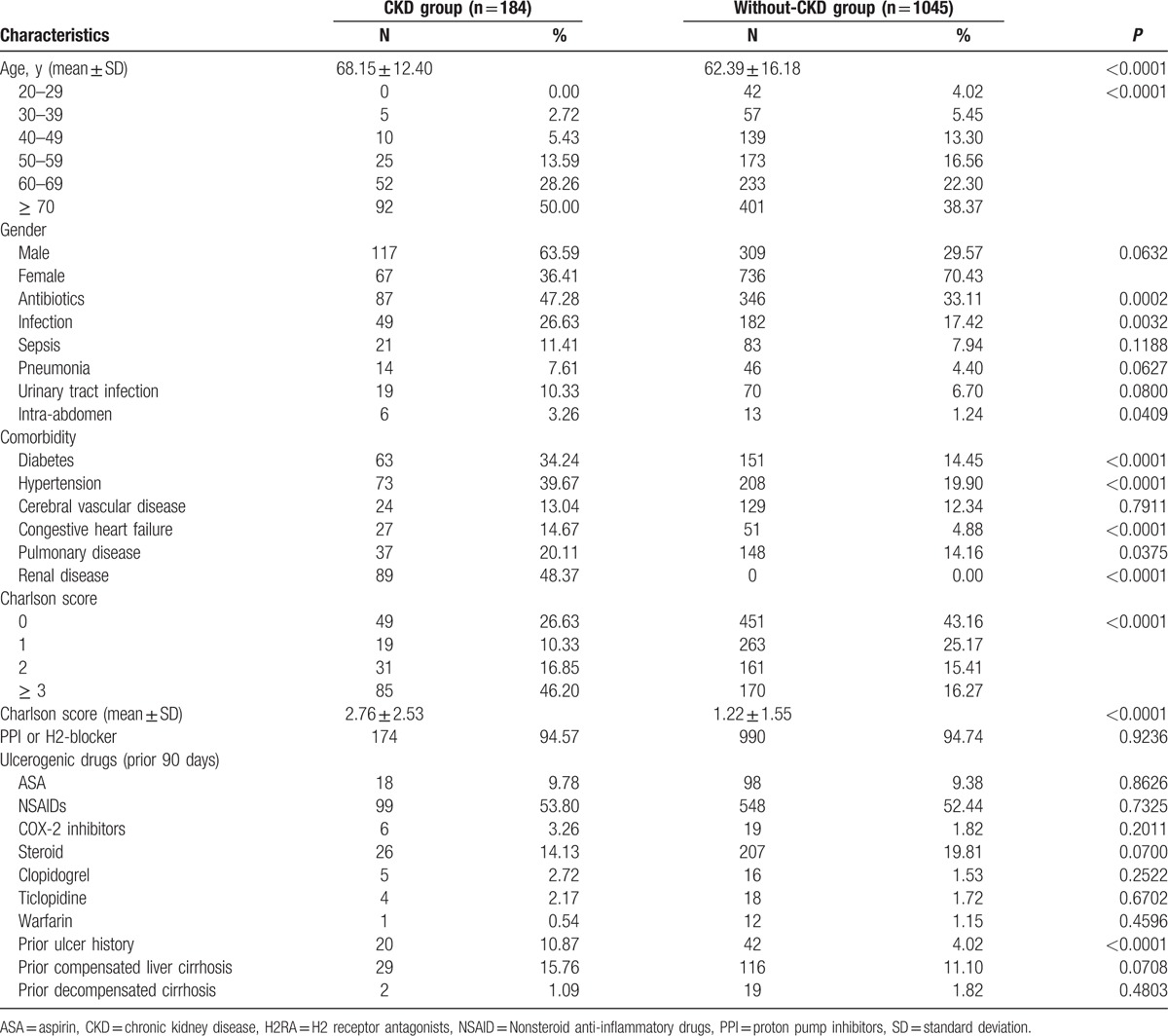
Clinical characteristics of patients.

**Figure 2:**
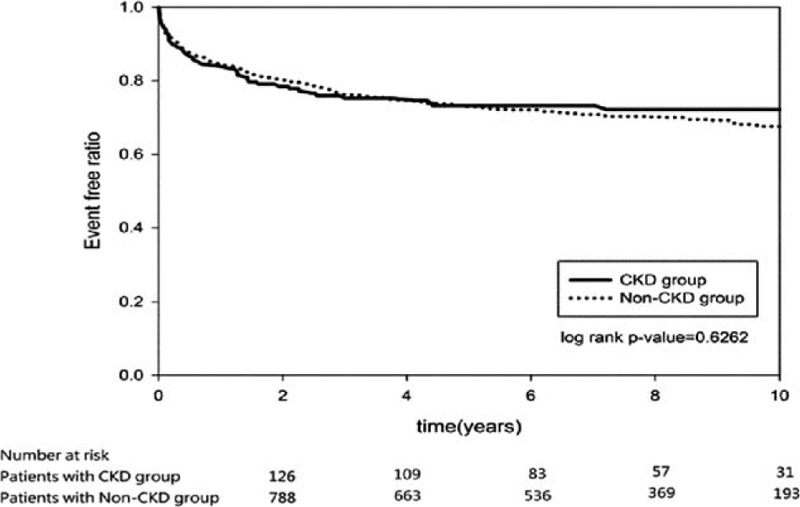
Kaplan–Meier curve of rebleeding after initial endoscopic hemostasis (10-year follow-up period).

**Figure 3:**
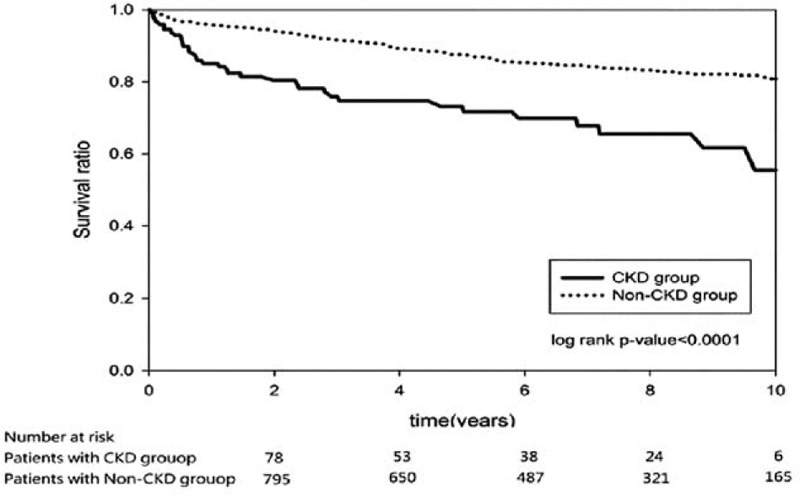
Kaplan–Meier curve of mortality rate after initial endoscopic hemostasis (10-year follow-up period).

Table 2 shows the outcome of the 2 groups. Higher rebleeding rates (patients needed repeat endoscopic therapy; 11.96%, vs 6.32%, *P* = 0.0062), death rates (8.7%, vs 2.3%, *P* < 0.0001), and hospitalization cost (US$ 5595 ± 7200 vs US$2408 ± 4703, *P* < 0.0001), and longer length of hospital stay (19.6 ± 18.3 vs 11.2 ± 13.1, *P* < 0.0001) were observed in patients with CKD than in patients without CKD. The death rate in the CKD group was also higher than that in the non-CKD group during the long-term follow-up period after discharge (22.02%, vs 13.71%, *P* = 0.0050). The independent risk factor for rebleeding during hospitalization was age (OR, 1.02; *P* = 0.0063; Table 3), whereas the risk factors for death were CKD (OR, 2.37; *P* = 0.0222), shock (OR, 2.99; *P* = 0.0098), and endotracheal intubation (OR, 5.31; *P* < 0.0001; Table 4). The hazard ratio of rebleeding risk for aspirin users after discharge over long-term follow-up period was 0.68 (95% CI 0.45–0.95, *P* = 0.0223; Table 5). On the other hand, old age (*P* < 0.0001), CKD (*P* = 0.0090), diabetes (*P* = 0.0470), and congestive heart failure (*P* = 0.0013) were the independent risk factors for death after discharge over the long-term follow-up period (Table 6). No significant difference was found between the 2 groups with respect to the rate of recurrent PUB (*P* = 0.6262; Fig. 2), but the mortality rate was significantly different in the CKD group (*P* < 0.0001; Fig. 3) in the Kaplan–Meier curve of the 10-year follow-up period after initial endoscopic hemostasis therapy.

**Table 2:**
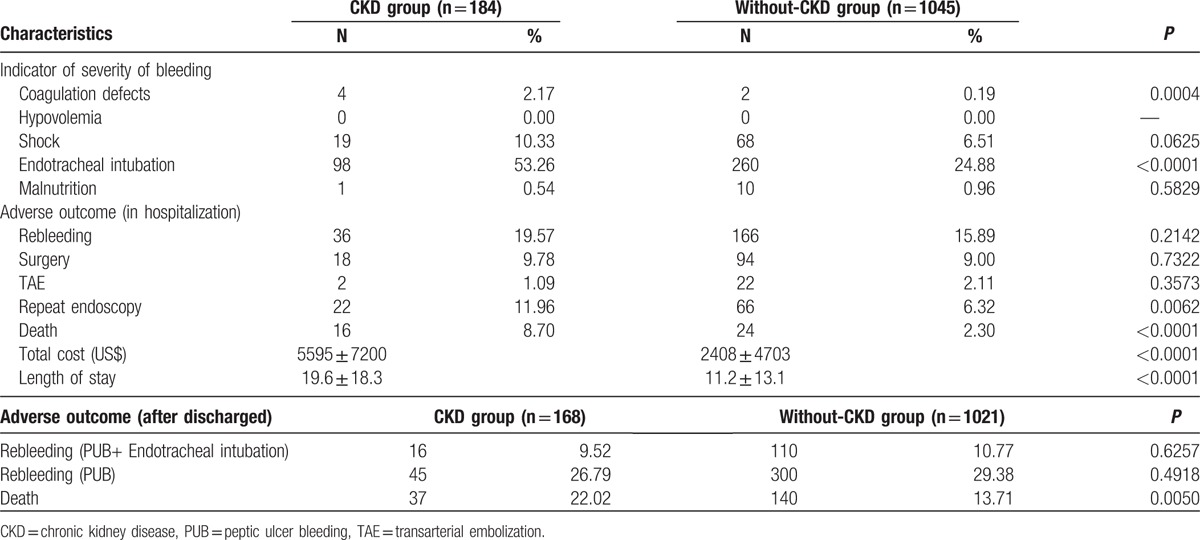
Outcomes of 2 groups.

**Table 3:**
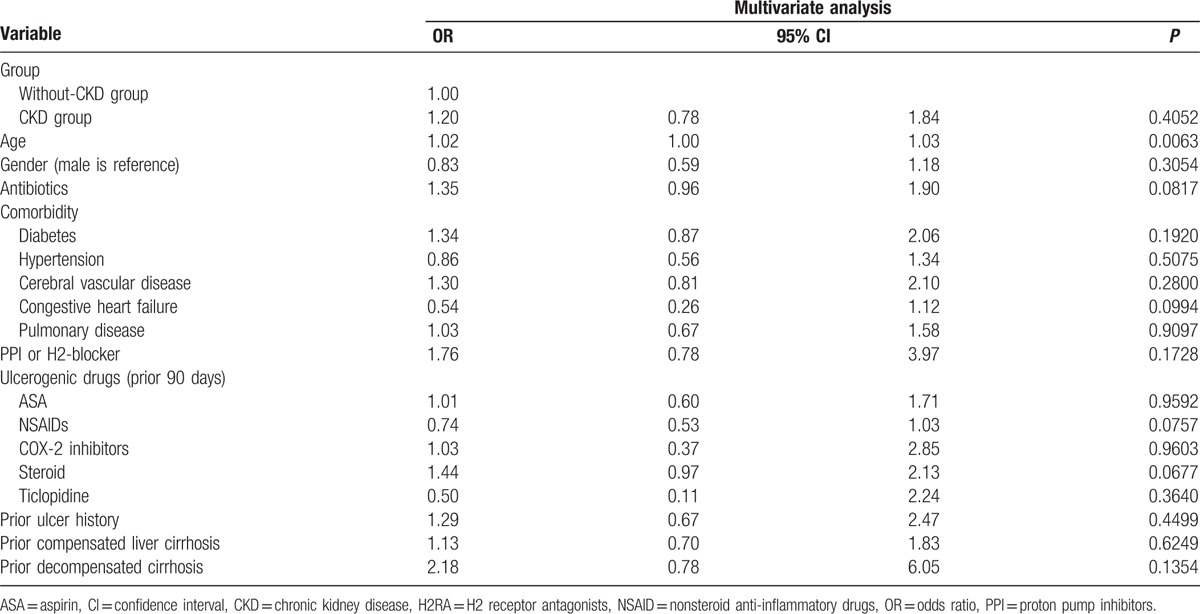
Multivariate analysis for rebleeding in index hospitalization.

**Table 4:**
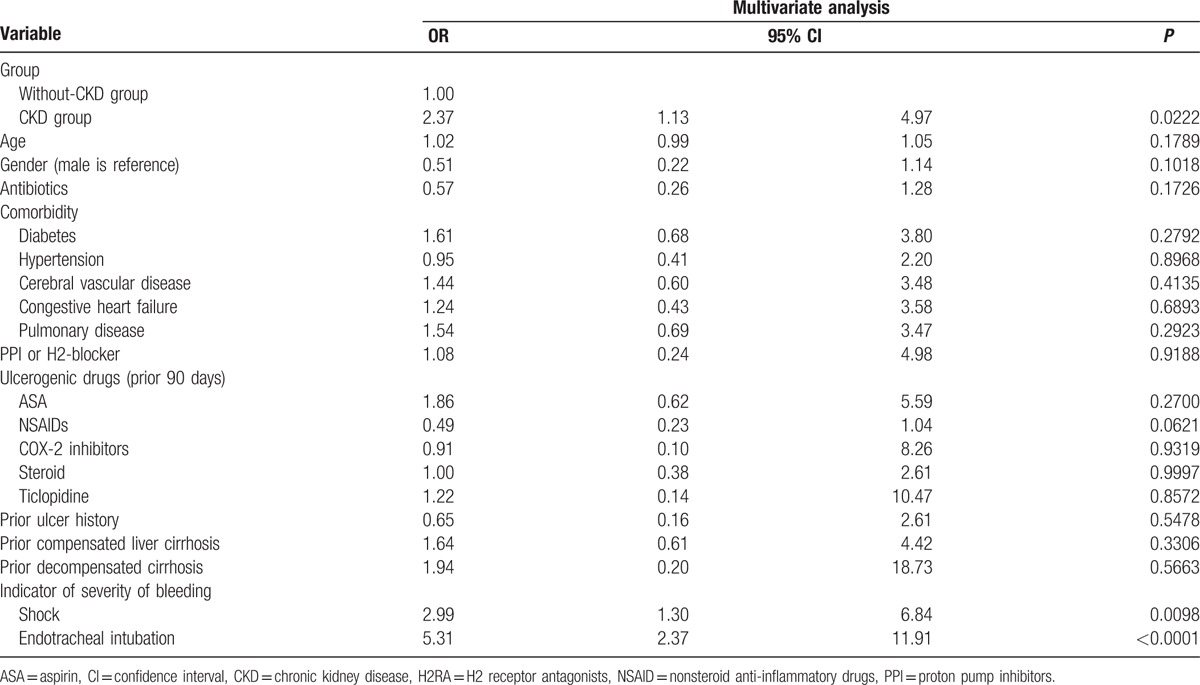
Multivariate analysis for death in index hospitalization.

**Table 5:**
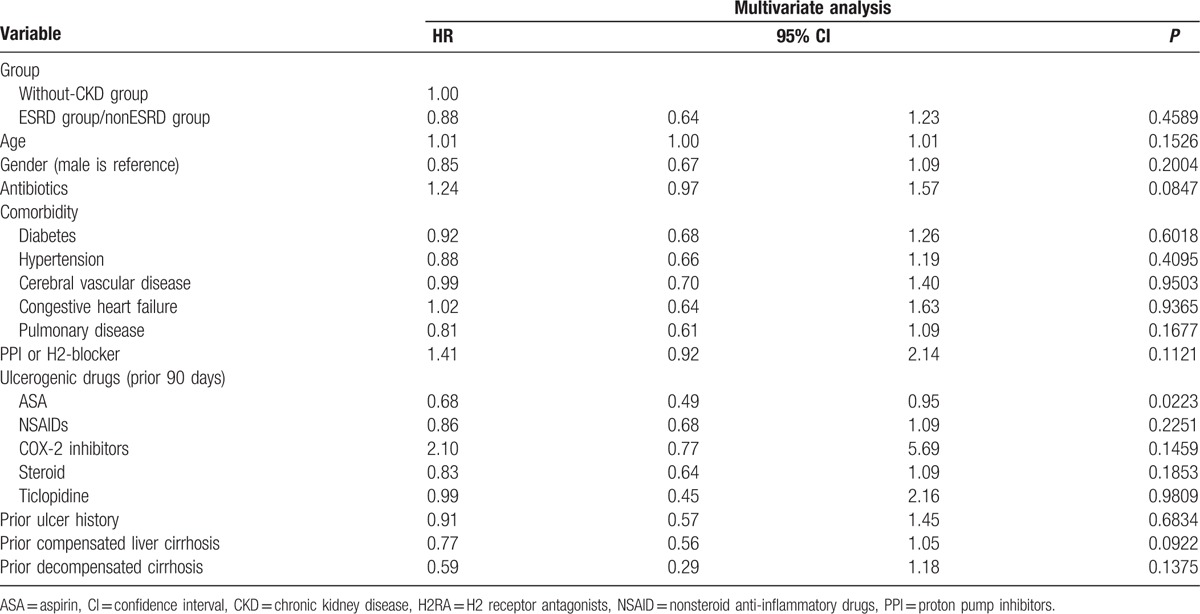
Independent risks for rebleeding after discharge.

**Table 6:**
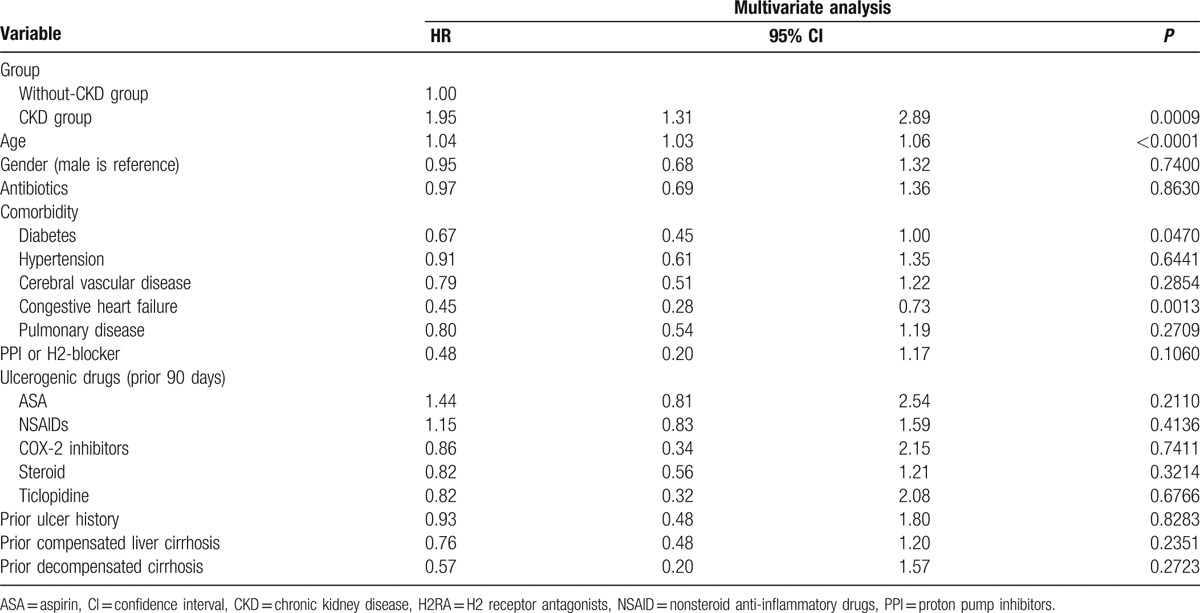
Independent risks for death after discharge.

## 4 Discussion

In this population-based case–control study using the Taiwan NHRID, the risk factors were identified, which influenced the outcomes of patients with CKD and PUB after initial endoscopic hemostasis, and the long-term outcome over a 10-year follow-up period were determined and compared between the CKD and non-CKD patients. The independent risk factor for rebleeding during hospitalization was age, whereas the risk factors for death were CKD, shock, and endotracheal intubation. Ten-year follow-up data after discharge showed no significant difference between the 2 groups with respect to the rate of recurrent PUB, but the mortality rate was significantly different in the CKD group.

Our previous case–control study showed that patients with ESRD experienced higher in-hospital recurrent bleeding rates (ESRD vs CKD vs control: 43% vs21% vs 12%; *P* < 0.001) after endoscopic hemostasis therapy for high stigmata ulcer.^[12]^ The present study increased the study cohort numbers by using the NHRID of Taiwan. In-hospital patients with CKD and PUB had higher recurrent bleeding, infection, and mortality rates after initial endoscopic therapy, and the need for second endoscopic therapy. This was similar to Liang's report that a higher incidence rate of PUB, more than 10 times, was observed in patients with CKD than that in patients without CKD, especially in elderly patients who have higher recurrent bleeding rate than younger patients.^[3]^ Many possible explanations are reported for more bleeding events in patients with CKD, especially those in the uremic stage. Uremic platelet function impairment was a potential cause of the higher risk for ulcer bleeding complications.^[14]^ Uremic platelet dysfunction involves the interaction of von Willebrand factor with platelet membrane glycoproteins Ib and IIb to IIIa, which is not normalized after dialysis.^[15]^ On the other hand, uremic platelet dysfunction could also be caused by prolonged exposure to the artificial surface of the dialyzer membrane, resulting in platelet exhaustion, or the use of heparin during hemodialysis, which could leak from the catheters.^[16]^ Therefore, uremic patients were susceptible to more bleeding events, which is similar to our in-hospital patients with CKD and PUB.

Furthermore, patients with CKD had prolonged hospital stays and increased mortality rates, as reported in some studies.^[14,15]^ CKD and ESRD metabolic alterations of uremia favor bacterial overgrowth in the gut and increase the translocation of living bacteria and bacterial components, which may result in systemic inflammation and acquired immunodeficiency. This dysbiosis may then result in cardiovascular disease, body wasting, and infections, and even causes deaths.^[17,18]^ Vitamin D deficiency is a common problem in patients with CKD stages III to V.^[19,20]^ Vitamin D is a potent immunomodulator, wherein monocytes and macrophages exposed to a bacterial lipopolysaccharide upregulate the vitamin D receptor gene, which results in the synthesis of cathelicidin, a peptide capable of destroying bacterial agents.^[21–23]^ Therefore, it is rational that patients with vitamin D deficiency could be susceptible to increased death rates in the uremic cohorts. In our study, incidence of hospital infections in the CKD group were higher than those in the non-CKD group (26.63%, vs 17.42%, *P* = 0.032). This could explain why CKD was the independent risk factor for mortality in index of hospitalization (OR, 2.37; *P* = 0.0222) or even after discharge (OR, 1.95; *P* = 0.0090).

The hazard ratio of recurrent bleeding risk for aspirin users after discharge over the long-term follow-up period was 0.68 (95% CI 0.45–0.95, *P* = 0.0223) in the present study. Meanwhile, more patients were found to have PUD history in the CKD group than in the non-CKD group (10.87% vs 4.02%, *P* < 0.0001). Based on the Taiwan National Health insurance policy, doctors should shift aspirin to clopidogrel in patients with a history of PUD. It was reported that gastrointestinal hemorrhage was significantly less frequent in patients who use clopidogrel than in those who were prescribed aspirin (1.99% vs 2.66%, *P* < 0.002).^[24]^ On the other hand, a phenomenon of gastric adaptation to aspirin exists in patients with long-term aspirin use. Graham and colleagues observed that gastric erosions or hemorrhage occurred within 24 h after aspirin administration.^[25]^ However, injury was maximal within the first 3 days and then subsequently lessened. The degree of mucosal injury became markedly less severe after 7 days of aspirin use compared with that observed after 1 day of therapy. It was also observed that deoxyribonucleic acid recovery (a marker for cellular exfoliation and regeneration) increased significantly after aspirin use. Therefore, gastric adaptation to aspirin chronic injury could involve an increase in cellular regeneration.^[26]^ Alderman et al also reported an increase in the level of mucosal regenerating protein (RegI) during the development of the adaptation process. It was maintained during subsequent aspirin dosing, and returned to baseline levels once dosing had ceased and stopped the gastric adaptation process.^[27]^ However, *H pylori* infection could also impair the gastric adaptation process to aspirin, and eradication of the bacteria would restore this process.^[28,29]^

The present study observed that the rate of recurrent PUB was not different between the CKD and non-CKD groups in the 10-year follow-up period. The bottom line is, as high as 94.57% of the CKD cohort was long-term PPI or H2-blocker users. Long-term H2 blocker use for high-risk bleeding patients would reduce the annual recurrence of PUB from nearly 70% to approximately 25%.^[30]^ Further studies are required to confirm the protective role of long-term use of H2 blockers for PUD.

Several limitations of this study should be recognized. First, this retrospective analysis was dependent on the completeness of documentation of the ICD code in the index of hospitalization, especially the ICD record of CKD. The definition of CKD depended on the estimated glomerulofiltration rate calculated from age, sex, and serum creatinine level by using an isotope dilution mass spectrometry traceable equation. However, most doctors defined CKD based on plasma creatinine levels only. The CKD population could be underestimated. Second, the data regarding *H pylo*ri infection in this study was obtained when a record of *H pylori* eradication therapy was identified during or after the index hospitalization, such as PPIs or H2 receptor antagonists (H2RA), plus clarithromycin or metronidazole, plus amoxicillin or tetracycline, with or without bismuth, and other regimens. Hence, we cannot analyze the effect of *H pylori* and PUB in patients with CKD. Third, nearly 100% of patients were administered with PPI or H2RA for bleeding, which makes estimating the protection effect difficult in PUB.

In conclusion, hospitalized patients with CKD and PUB after endoscopic therapy had higher recurrent bleeding, infection rate, and mortality rate and needed second endoscopic therapy. Age was the independent risk factor for recurrent bleeding during hospitalization. After being discharged with a 10-year follow-up period, nonaspirin user was significant factor for recurrent bleeding. Given the long-term H2 blocker use and possible adaptation mechanism for aspirin, more research relating to peptic ulcer risk in the CKD population is warranted.
